# Dynamic evaluation of corneal cross-linking and osmotic diffusion effects using optical coherence elastography

**DOI:** 10.1038/s41598-024-67278-1

**Published:** 2024-07-18

**Authors:** Matteo Frigelli, Philippe Büchler, Sabine Kling

**Affiliations:** ARTORG Center for Biomedical Engineering Research, University of Bern, Bern, Switzerland

**Keywords:** Optical coherence elastography, Corneal cross-linking, High-resolution dynamical analysis, Corneal preservation media, Osmotic effects, Biomedical engineering, Imaging and sensing

## Abstract

Dynamic deformation events induced by osmosis or photochemical stiffening substantially influence geometrical and mechanical assessments in post-mortem corneas, therefore need to be carefully monitored in experimental settings. In this study, we employed optical coherence elastography (OCE) to quantify dynamic deformation processes at high resolution in freshly enucleated porcine corneas. Osmotic effects were studied by immerging n = 9 eyes in preservation media of three different tonicities. Dynamic processes underlying corneal cross-linking (CXL) were studied by subjecting n = 6 eyes to standard Dresden treatment, while three control groups were used. The entire procedures were performed under an OCE setup during up to 80 min, acquiring a volumetric scan every 20 s. Changes in OCE-derived axial deformations were incrementally calculated between consecutive scans. Preservation conditions had a strong influence on the observed strain patterns, which were consistent with the tonicity of the medium (swelling in hypotonic, deswelling in hypertonic environment). In the CXL group, we observed deswelling of the anterior stroma 10 min after starting the UV irradiation, which was not observed in any control group (*p* = 0.007). The presented results proved OCE to be a valuable technique to quantify subtle dynamic biomechanical alterations in the cornea resulting from CXL and preservation solutions.

## Introduction

Over the past two decades, optical coherence tomography (OCT) has been widely used in ophthalmology to image different ocular tissue structures and to aid in the diagnosis and monitoring of various ocular diseases such as glaucoma^1,2^, cataract^3^ and keratoconus^4^. The remarkable clinical efficacy^5^ of this technique can be attributed to two key factors: it is non-invasive and it offers much higher axial and lateral resolution (~ 5 and 20 µm, respectively) compared to other medical imaging systems such as ultrasound and magnetic resonance imaging (MRI)^6^.

Building on the success of OCT, optical coherence elastography (OCE) has emerged as a powerful extension of this imaging modality, with the first pioneering study published in 1998^7^. OCE measures the deformations that occur between two consecutive OCT scans when the tissue is subjected to a mechanical stimulus. By analyzing the changes in amplitude and phase of successive OCT scans, which are described as complex numbers, OCE enables tracking and measurement of tissue displacements and deformations, providing valuable information about tissue stiffness and mechanical properties^8,9^. Various mechanical stimuli have been proposed to induce tissue deformation in combination with OCE imaging. They can be divided into contact (planar compression, quasi-static indentation) and non-contact (micro air pulse, laser pulse, ambient pressure modulation) approaches. OCE can also be performed using passive approaches, i.e. without externally triggered stimuli, but using the natural movements caused by intrinsic physiological forces^10,11^. With respect to the anterior segment of the eye, OCE has been widely applied *ex-vivo* and has provided useful insights into the biomechanics of the sclera^12^, lens^13^, limbus^14^, and cornea^15,16^. Only few OCE in-vivo setups have also been proposed, and shown to be effective in quantifying the induced deformations in the cornea^17–22^ and the lens.

OCE has already been used to investigate deformation processes caused by osmotic processes^23^. Alexandrovskaya et al. analyzed the osmotic strain dynamics in cartilage samples and demonstrated the applicability of OCE for the time-dependent quantification of such deformations^24^. The authors applied optical clearing agents to the samples, which induced osmotic deformations leading to axial strain magnitudes between 10^−4^ and 0.4 [–], which could be measured with a spatial resolution of 50 µm both laterally and axially and a temporal resolution of 1 s. Lawman and colleagues^25^ employed deformation velocity imaging (DVI) to visualize and quantify the mechanism of de-swelling in human corneal samples by examining the effects of the presence of endothelium on the osmotic flow with a temporal resolution of 1 min. These studies have shown that OCE can efficiently characterize the dynamic osmotic processes at high resolution and provide temporally and spatially resolved dynamic quantification of osmotically induced deformations that would not be possible with other imaging techniques.

Hydration severely affects the mechanical properties of the cornea, with both the longitudinal bulk modulus and the tangential elastic modulus of the tissue decreasing with swelling^26,27^. External conditions (e.g. room temperature, preservation media, time since the collection of the samples) can rapidly change the level of hydration of the corneal samples, e.g. causing it to swell, thus affecting its mechanical properties. In order to restore the physiologic thickness of the tissue, it is common to deswell the cornea using a hypertonic solution (e.g. Dextran in different percentages) to remove the excess water by a modified osmotic pressure. As it has been shown that the rate of deswelling can damage the tissue, efforts have been made to optimize the deswelling process^28,29^. In this context, quantifying and controlling the dynamic processes in the cornea at high resolution could provide valuable insights into the time-dependent osmotic effects to which the tissue is exposed in the experimental conditions.

The degree of hydration is also a crucial factor to monitor during *ex-vivo* corneal cross-linking (CXL), as the outcome of the procedure has been shown to depend on the initial degree of hydration of the tissue^30^. CXL is a well-established treatment to stop the progression of keratoconus and corneal ectasia by stiffening the cornea, by inducing the formation of cross-links in the extracellular matrix^31,32^. Three major factors are required for CXL to occur: riboflavin, UVA light exposure and an oxygen-rich environment to trigger the formation of reactive oxygen species. These reactive oxygen species subsequently facilitate the creation of crosslinks within the extracellular matrix^32,33^. The kinetics of oxygen consumption during CXL treatment have been previously investigated, with authors describing different chemical pathways that promote cross-linking between corneal collagen at the molecular level^34^. For the oxidative pathway, a predominant type II mechanism has been described at high oxygen concentration, whereas the photochemical reaction at complete oxygen deprivation is consistent with a predominantly anaerobic type I mechanism^35,36^.

When applied *ex-vivo* to study changes in corneal biomechanical properties after CXL, OCE showed an increase in elastic anisotropy and in Young modulus after the procedure^37,38^, as well as an altered strain distribution that was restricted to the area where CXL was performed^39^. OCE also showed an increase in shear modulus in different posterior scleral regions after scleral CXL^40^ in porcine eyes. However, to our knowledge, there is no previous study investigating the dynamic mechanical processes induced by CXL during treatment, neither with OCE nor with other imaging techniques.

The present study has two objectives: first to evaluate the suitability of OCE for the dynamic assessment of corneal deformation *ex-vivo* by testing the tissue under different preservation conditions. Second, to use this method to evaluate dynamic changes during and after CXL treatment.

## Materials and methods

**Figure 1:**
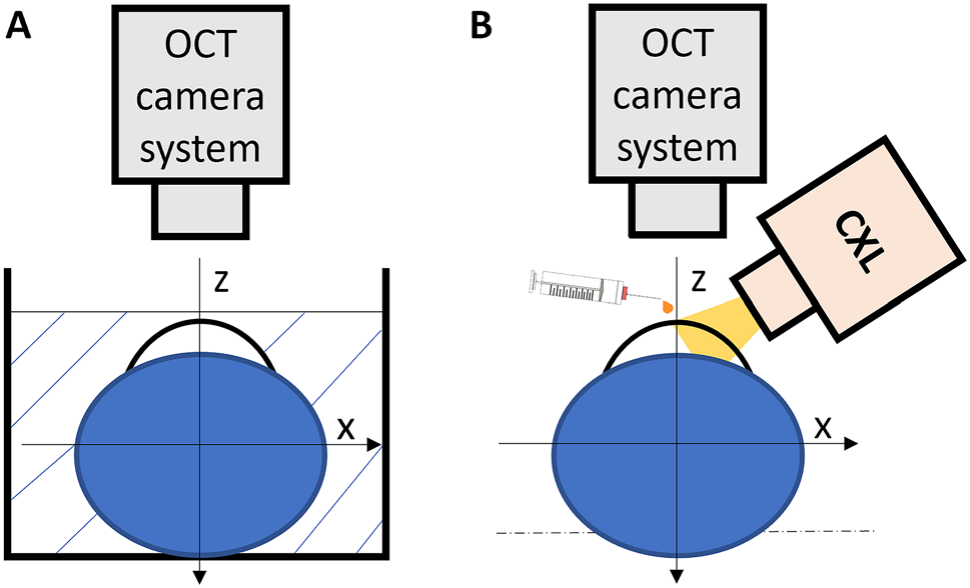
Schematic representation of the two experimental setups used for the study. (**A**) was used to evaluate the dynamic changes induced on the cornea by different preservation media, with the globe immersed in it; (**B**) was used to investigate the active changes induced by CXL during and after treatment.

Two different experimental OCE protocols (Fig. 1) were developed to investigate (i) the dynamic mechanical changes in the cornea induced by different preservation media and (ii) the active mechanical effect induced by the CXL procedure. Both protocols were characterized by the absence of any additional external mechanical stimulus applied to the tissue.

### OCT system

The same spectral domain OCT system^16^ and identical post-processing algorithms were used in both protocols. The device operates at a wavelength of 877.8 nm, a bandwidth of 62.5 nm, and an output power of 1.62 mW. For each measurement, a volumetric scan (C scan) of a 12 × 12 mm was acquired in the central cornea, consisting of a stack of 100 2D-images (B scans). Each B-scan has an axial resolution of 3.26 μm (in the corneal tissue) and a lateral resolution of 12 μm (consisting of 1000 A-scans). The typical acquisition duration for a C-scan was 11 s. The OCT software was programmed to acquire C scans at a specific frequency during the experiments.

### OCE strain calculation

Subsequent OCT C-scans were compared to quantify the relative mechanical deformation of the cornea in the direction of the optical axis, z, using a phase-sensitive deformation tracking algorithm that has been previously described in detail^16^. The algorithm was implemented using custom-developed MATLAB routines (Massachusetts: The MathWorks Inc., 2019). Briefly, the tissue displacement is proportional to the phase difference between two consecutive OCT scans, which is represented by the angle of the complex cross-correlation ∠C(z, x). The axial displacement U(z, x) (m) is therefore given by the relationship^41,42^:

1 $$U\left( {z,x} \right) = { }\frac{{\lambda_{mean} \cdot \angle C\left( {z,x} \right)}}{4\pi n}$$

where λ_mean_ = 877.8 nm is the central wavelength and n = 1.375 [–] is the refractive index. The amplitude-weighted complex cross-correlation W(z, x, y) calculated between the two OCT interference signals, which are both recorded at the axial position z and the lateral position x, of the first (C_1_) and second (C_2_) OCT C-scan:

2 $$W\left( {z,{ }x,y} \right) = \frac{{\mathop \sum \nolimits_{{j = - w_{z} }}^{{w_{z} }} \mathop \sum \nolimits_{{k = - w_{x} }}^{{w_{x} }} C_{1} \left( {{\text{z}} + {\text{j}},{\text{ x}} + {\text{k}},{\text{y}}} \right){ } \cdot C_{2}^{*} \left( {{\text{z}} + {\text{j}},{\text{ x}} + {\text{k}},{\text{y}}} \right)}}{{\left| {\mathop \sum \nolimits_{{j = - w_{z} }}^{{w_{z} }} \mathop \sum \nolimits_{{k = - w_{x} }}^{{w_{x} }} C_{1} \left( {{\text{z}} + {\text{j}},{\text{ x}} + {\text{k}},{\text{y}}} \right){ } \cdot C_{2}^{*} \left( {{\text{z}} + {\text{j}},{\text{ x}} + {\text{k}},{\text{y}}} \right)} \right|}}$$

Here, w_z_ = 3 and w_x_ = 3 (pixels) represent the dimensions of the phase-processing window used.

The axial strain ${\varepsilon }_{zz}$ [–] is calculated as the axial gradient of the displacement, according to the formula ^41^:

3 $$\varepsilon_{zz} = { }\frac{{{\text{d}}U}}{{{\text{d}}z}} = { }\frac{{\lambda_{mean} \cdot \angle R}}{4\pi n\delta }$$

where $\delta$=4.48 μm is the axial sampling unit, ∠R is the angle of a second complex cross-correlation $R\left(z, x,y\right)=\sum_{j=-{w}_{z}}^{{w}_{z}}\sum_{k=-{w}_{x}}^{{w}_{x}}W\left(\text{z}+\text{j},\text{x}+\text{k},\text{y}\right) \cdot {W}^{*}\left(\text{z}+1+\text{j},\text{ x}+\text{k},\text{y}\right)$, and w_z_ = 3 and w_x_ = 3 (pixels). Due to the phase-processing windows applied in both cross-correlations, the axial and lateral resolutions of the resulting strain maps (39 and 144 μm, respectively) were correspondingly lower than the original resolutions of the structural images.

To visualize the depth dependent deformation over time, the OCE-derived axial strain data from the central B-scan were extracted for the 40 central A-scans (spanning across 448 µm along the x axis), averaged and plotted for each time frame throughout the experiment.

To compare anterior and posterior corneal deformation over time, the OCE-derived axial strains calculated between subsequent volumetric scans were averaged over the central 4 × 0.48 mm region, for the anterior and posterior 400 µm of corneal thickness, respectively, and finally accumulated.

#### Dynamic effects induced by different preservation media

A total of 9 freshly enucleated porcine eyes were tested no later than 16 h after collection from the local slaughterhouse. The eyeballs were fixed in a holder and immersed in a beaker filled with a preservation medium (Fig. 1A). Using OCT, the apex of the cornea was positioned less than 0.5 mm from the liquid surface. This distance was chosen to minimize signal loss of the OCT light in the central cornea. A volumetric scan was acquired every minute for 45 min. We tested the dynamic effects of 3 different preservation media, characterized by different osmolarities: (i) phosphate buffered saline (PBS, Sigma-Aldrich, Merck Group, Burlington, MA), containing 137 mM/L NaCl, (ii) a solution of minimum essential medium (MeM, Sigma-Aldrich) and 5% Dextran and (iii) a solution of MeM and 7% Dextran. Table 1 summarizes the different osmolarities of the solutions used in the study. The osmolarity of the porcine cornea was taken from the literature^43^. Each medium was tested on n = 3 samples and measurements were performed at room temperature.

**Table 1:** Osmolarity ranges for the different solutions used in the study.

	PBS	MeM	MeM dextran 5%	MeM dextran 7%	Porcine cornea^43^
Osmolarity (mOs/kg)	275–304	270–310	369–409	409–449	329 ± 61

#### Dynamic effects induced by CXL treatment

A total of 24 freshly enucleated porcine eyes were tested within 16 h of collection from the local slaughterhouse. The eyes were de-epithelialized and divided into four groups, which were treated as follows: (1) PBS-only group (n = 6): PBS drops every 5 min for 50 min; (2) UV-only group (n = 6): PBS drops every 5 min for 20 min, followed by irradiation with UVA light for 30 min, at a irradiance of 3mW/cm^2^, with the eye hydrated with PBS every 5 min during the irradiation period; (3) RIBO-only group (n = 6): drops of 0.1% riboflavin (Streuli Pharma AG, Switzerland) in PBS every 5 min for 50 min; (4) CXL group (n = 6), standard Dresden CXL treatment: drops of 0.1% riboflavin in PBS solution every 5 min for 20 min before UV irradiation and every 5 min during UV irradiation (30 min, 3mW/cm^2^ irradiance). After the group-specific protocol was completed, an additional 30 min period with PBS drops every 5 min was performed in all groups to investigate any delayed responses.

The entire procedure including the final 30-min PBS administration, (80 min in total) was performed at room temperature under OCT scanning (Fig. 1B), with the globe placed in a custom-made holder to avoid any unwanted movement while volumetric C-scans were acquired every twenty seconds.

### Statistical methods

Statistical analyses were performed using GraphPad Prism 8.0.1 (Graph-Pad Software Inc, La Jolla, Calif). Continuous variables were expressed as means (± standard deviation) or medians (Q1–Q3).

Samples belonging to groups 1–4 were compared by analysing the differences between the slopes of the strain–time curves at the different phases of the experiment. Paired or unpaired t-tests were performed to test for statistically significant differences between the slopes of the curves at various time points, with differences considered statistically significant if the *p*-values were smaller than 0.05.

## Results

### Dynamic effects induced by different preservation media

**Figure 2:**
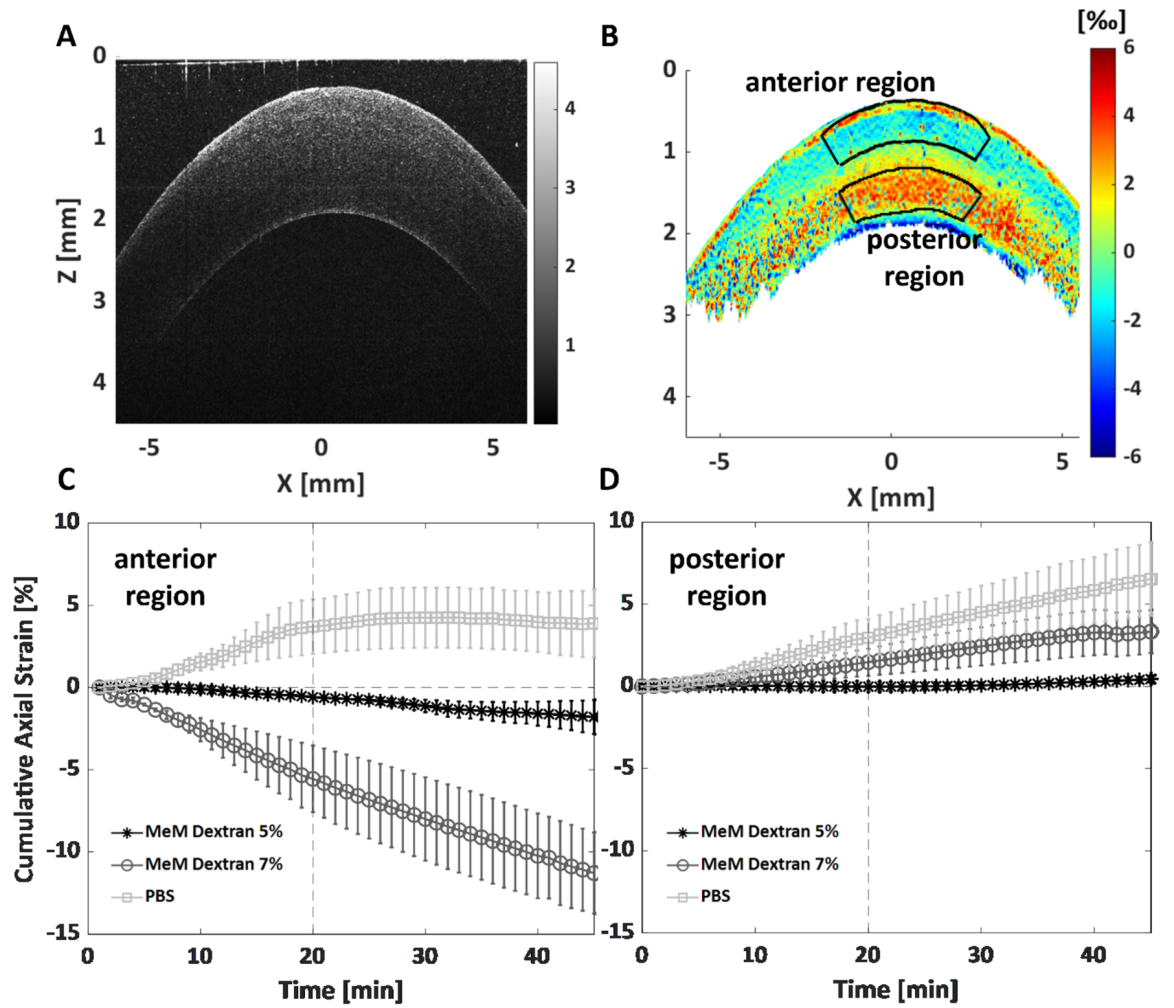
Effect of the three different preservation media on the anterior stroma measured via cumulative axial strain. (**A**) Structural OCT image of the central B-scan; (**B**) corresponding axial strain map on a sample preserved in MeM Dextran 5% at minute 40. The area outlined in black indicates the anterior region that was averaged to calculate the axial strain in the anterior stroma; (**C**) cumulative axial strain in the anterior stroma as a function of time for the entire experiment duration (45 min) for the three different preservation media; (**D**) cumulative axial strain in the posterior stroma as a function of time for the whole experiment duration (45 min) for the three different preservation media. Data are expressed as mean ± standard deviation.

The effects of different preservation media on the strain in the anterior and posterior parts of the stroma are shown in Fig. 2. The time-dependent cumulative axial strain curves in the anterior region (Fig. 2B) show clear differences between the three different preservation media (Fig. 2C). During the first 20 min, samples in PBS showed an anterior stromal expansion of 3.7 ± 1.7%. The strain curve stabilized after 20 min when they reached osmotic equilibrium. After 45 min, the samples in PBS showed a final cumulative strain value of 3.9 ± 2.1%. Corneas immersed in MeM Dextran 7% solution showed an axial shrinkage of the anterior stroma of − 5.6 ± 2.0% and − 11.3 ± 2.5% after 20 and 45 min, respectively. On the other hand, MeM Dextran 5% solution resulted in minimal deformations compared to the other two preservation media, with the anterior stroma reaching a final cumulative strain value of -1.8 ± 1.0% after 45 min, therefore maintaining the tissue’s hydration stable. Differences between the preservation media were also observed for the posterior stroma in the time-dependent cumulative axial strain (Fig. 2D). We observed posterior swelling under all conditions, which is related to the fact that the solution was applied exclusively to the anterior surface and not to the entire thickness of the sample. The samples in MeM Dextran 5% showed only minimal changes, with an axial strain value of − 0.01 ± 0.3% after 20 min and a final value of 0.4 ± 0.2%. MeM dextran 7% and PBS samples showed a linear increase in axial strain over time, with values of 3.3 ± 1.3% and 6.4 ± 2.2% respectively after 45 min, for the two media. The expected osmotic phenomena induced by preservation media displaying different osmolarities (Table 1) were captured by the OCE system with high resolution.

**Figure 3:**
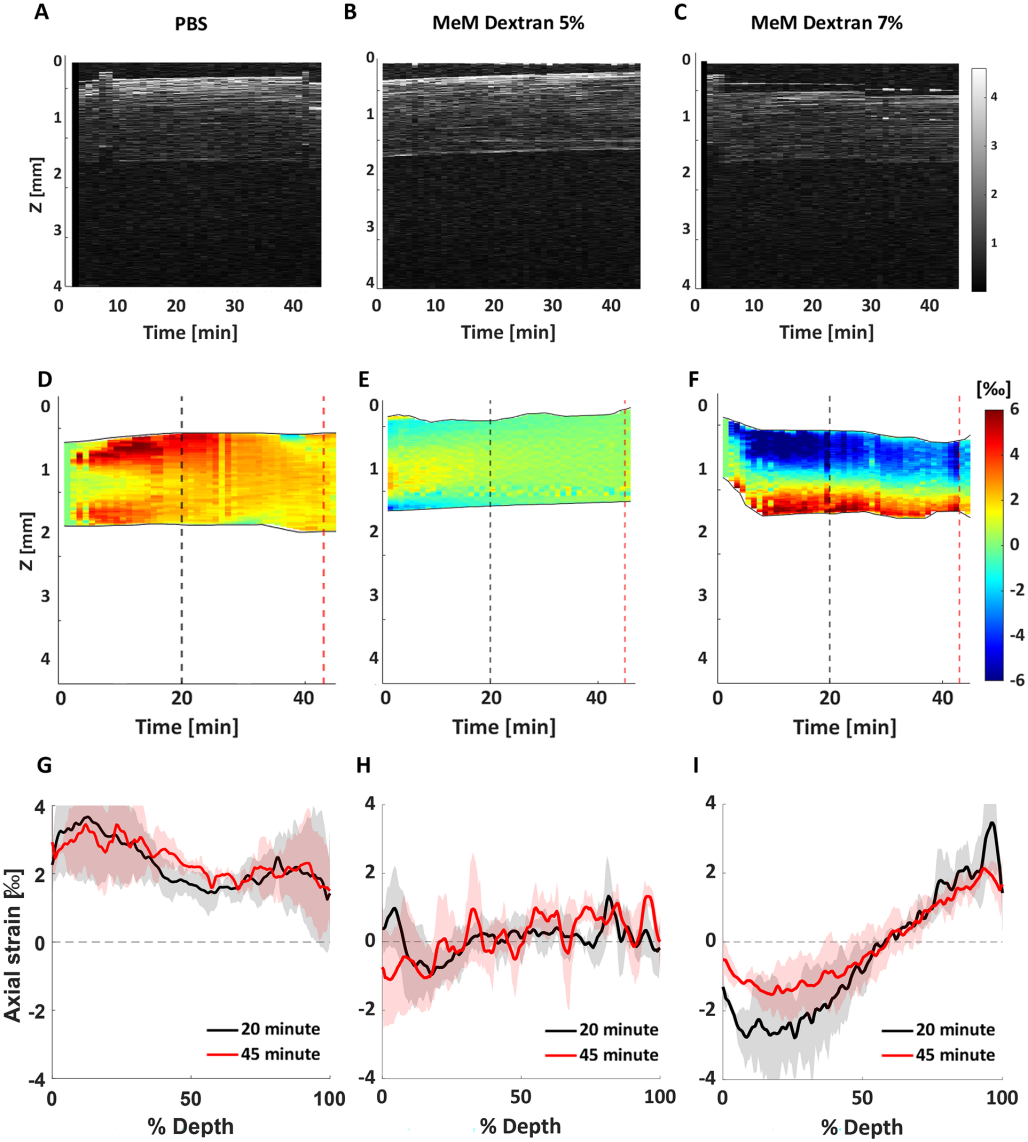
Dynamic quantification of the depth-dependent effect of different preservation media on the cornea: PBS (**A**, **D**, **G**), MeM Dextran 5% (**B**, **E**, **H**), and MeM Dextran 7% (**C**, **F**, **I**). Top row: structural image of the averaged 0.5 mm central region as a function of time; middle row: corresponding axial strain maps; bottom row: axial strain profile as a function of corneal thickness at minute 20 and 45. The first two rows show representative samples, while the last row shows the average profile over n = 3 samples for the groups considered.

To better describe the dynamic mechanical effects induced by the different preservation media over the entire corneal thickness, the time-depth map for each preservation media is shown in Fig. 3. The PBS samples exhibited a uniform axial strain expansion over the entire corneal thickness across the entire experiment (Fig. 3D,G). After a slight expansion of 1.5‰ in the central cornea during the first 10 min, the sample stored in MeM Dextran 5% solution showed a largely uniform strain pattern throughout the thickness for the duration of the experiment, reaching osmotic equilibrium at around minute 30 (Fig. 3E). The MeM Dextran 7% sample (Fig. 3F) showed an expansive strain pattern in the posterior cornea, which was strongest at the beginning and decreased towards the end of the measurement. This mechanism contrasts with the shrinkage observed in the anterior region of the same sample, the region where the preservation solution was in direct contact with the tissue. Figure 3I shows the corresponding approximately linear change in axial strain from − 2.5‰ at the anterior surface to + 2.5‰ at the posterior surface, and a transition region with zero strain at approximately 55% of the thickness.

### Dynamic effects induced by CXL treatment

**Figure 4:**
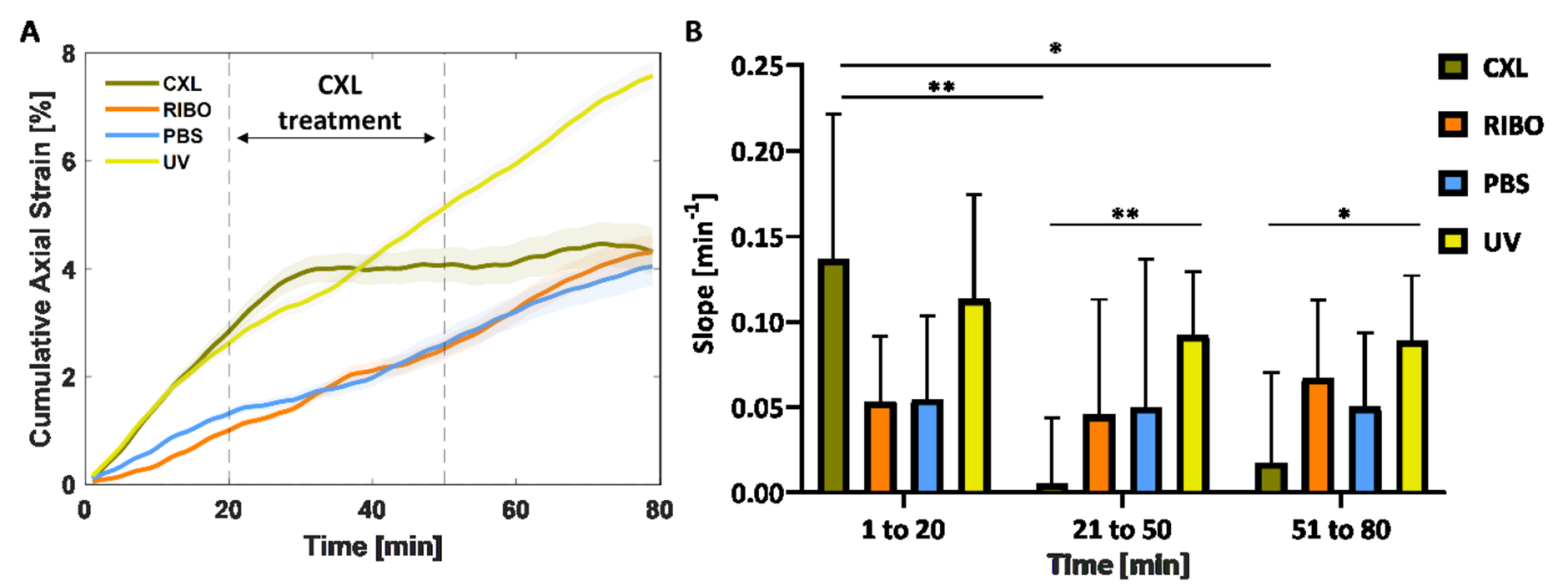
Dynamic effect of CXL treatment on the anterior cornea. (**A**) time-dependent cumulative axial strain in the anterior stroma during the experiment (80 min) for the four groups, expressed as mean ± standard error of the mean; (**B**) comparative analysis of the slopes of the curves from A in the three different time periods (before, during, after CXL). **.001 ≤ *p* ≤ .01; *.01 < *p* ≤ .05.

The curves of cumulative strain in the anterior stroma for the four different groups are shown in Fig. 4A. The three control groups (PBS-only, UV-only, RIBO-only) exhibited a consistent pattern, with the cumulative strain increasing steadily from the beginning to the end of the experiment (80 min). The CXL group demonstrated a comparable behavior up to 30 min. However, after that time point, which was 10 min after the start of the CXL treatment, the slope of the curve suddenly flattened out and showed an almost constant cumulative strain until the end of the experiment. To test the statistical significance, each experimental curve was divided into three sections: from minute 1 to 20 (before CXL treatment), from minute 21 to 50 (UV irradiation) and from minute 51 to 80 (after CXL treatment). Each section was fitted with a linear regression and the slopes *m* of the fitting lines were compared (Fig. 4B). The slope between minute 1 and 20 of the CXL group showed a statistically significant difference when compared to the second (m = 0.14 ± 0.08 min^−1^ vs m = 0.01 ± 0.04 min^−1^,* p* = 0.005) and the third segment of the curve (m = 0.14 ± 0.08 min^−1^ vs m = 0.02 ± 0.05 min^−1^, *p* = 0.019). Between minute 21 and 50, the CXL group was significantly flatter than the UV-only group (m = 0.01 ± 0.04 min^−1^ vs m = 0.09 ± 0.04 min^−1^, *p* = 0.007), and showed a trend towards significant differences compared to the PBS-only and RIBO-only groups (*p* = 0.081 and *p* = 0.090, respectively). A similar behavior was observed between minute 51 and 80, where the CXL group had a significantly flatter slope (m = 0.02 ± 0.05 min^−1^ vs m = 0.09 ± 0.04 min^−1^) compared to the UV-only group (*p* = 0.039).

**Figure 5:**
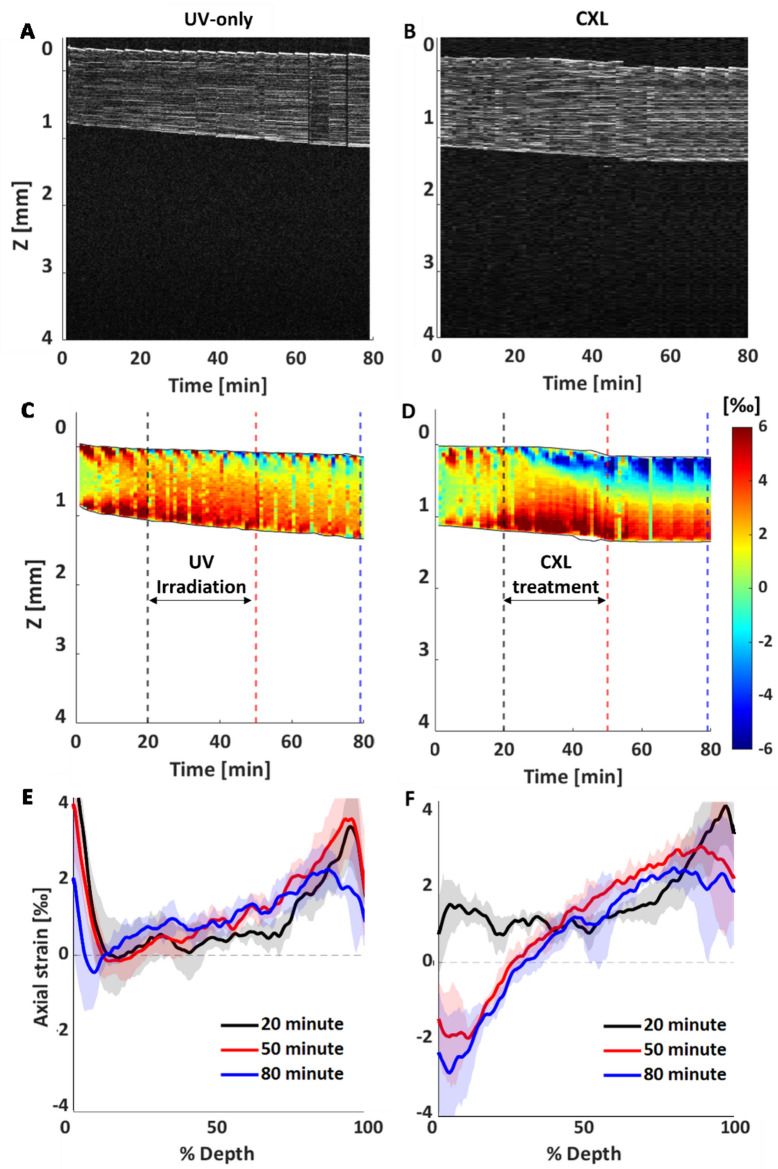
Dynamic quantification of the depth-dependent effect of CXL treatment on the cornea. Left column UV-only group, right column CXL group. (**A**–**B**) structural image of the central region (averaged across 480 µm) as a function of time; (**C**–**D**) corresponding axial strain maps. Artifacts (green vertical stripes) are due to the CXL procedure, in which 0.1% riboflavin in PBS solution was applied every five minutes, resulting in periodic loss of strain data; (**E**–**F**) axial strain variation across the depth of the sample at minute 20 (black line), 50 (red line) and 80 (blue line) for the groups considered. Error bars represent standard deviation. The first two rows show representative samples, while the last row shows the average profile over n = 6 samples for the groups considered.

The maps of axial strain as a function of time and depth (z) are reported for all groups: the comparison between the UV-only control and CXL groups is shown in Fig. 5 and in the Supplementary Figure S1. The control samples exhibited a constant positive axial strain, throughout the thickness and duration of the experiment, indicating swelling of the tissue. In the first 20 min, this mechanism was concentrated on the anterior and posterior corneal regions with axial strain values up to 4‰ strain (Fig. 5C vertical dashed black line and Fig. 5E). A similar behavior was observed in the pre-treatment phase of the CXL group (Fig. 5D vertical dashed black line and Fig. 5F). However, once the UV light was switched on, an area of negative strain formed, which gradually increased and eventually covered the anterior 200 µm of the cornea ten minutes after the start of the irradiation. This region continued to shrink steadily until the end of UV irradiation at minute 50, with this pattern remaining visible until the end of the experiment at minutes 80 (Fig. 5D). The depth map reported in Fig. 5F shows that the strain increases progressively at both the 50th and 80th minute, with mean values between -2.5‰ on the anterior and 4‰ on the posterior side, with the zero strain region located at 40% of the sample depth.

## Discussion

Osmotic effects in post-mortem corneal tissue influence its geometry and mechanical properties and must therefore be carefully controlled in the experimental setup. In this study, we presented an OCE-based system to quantify osmotically induced deformations with high spatial and temporal resolution. We investigated the effects of three different preservation media on the cornea and showed that varying the Dextran concentration by 2% significantly changes the mechanical strain in the anterior and posterior stroma. We then quantified for the first time the spatio-temporal strain generated in corneal CXL. We found that CXL causes a significant shrinkage of the anterior 200 µm starting ten minutes after the start of UV irradiation.

The measurement protocol used is non-invasive, fully automated, and allows the dynamic assessment of the mechanical deformations that occur in the cornea even when immersed in a preservation medium. The advantage of using a technique based on mechanical strain is that rigid body motions are automatically removed from the measurement and only the deformation of the tissue is measured. The high spatial resolution of the OCE enabled the visualization and quantification of local mechanisms that would not have been possible with an overall measurement such as the central corneal thickness. The central B-scan was chosen to minimize distortion and attenuation caused by the immersion of the sample in the liquid.

OCE has already been used to study corneal hydration^44,45^ and to evaluate the effects of CXL treatment on corneal stiffness^38^. Singh et al. showed an increase in Young’s modulus due to tissue dehydration and subsequent corneal thinning^45^. Vantipalli and colleagues measured the changes in corneal thickness and relaxation rate before and after CXL comparing the effects of isotonic and hypertonic riboflavin solutions. These authors showed that hypertonic CXL treatment leads to a reduction in stiffness that counteracts the increase in stiffness induced by CXL treatment^44^. While these studies provided valuable insight into the relationship between corneal thickness, hydration, and stiffness before and after CXL treatment, they did not examine the mechanical deformation the tissue undergoes during UV irradiation. The novelty of the present study is that the dynamic strain changes in the cornea in response to CXL treatment can be measured with high temporal resolution both during and after treatment.

Two different factors may affect the swelling of the cornea during CXL treatment: i) the osmotic pressure induced by the hydration media and the photosensitizer when applied to the sample, and ii) a possible effect exerted by the new cross-links created by the treatment itself, that could alter the osmotic balance in the anterior stroma and perhaps induce mechanical shrinking of the tissue. The objective of this study is to evaluate and measure the impact of CXL-induced dynamic effects, while excluding possible confounding factors such as osmotic swelling. Therefore, in the first part of the study, the osmotic pressure effects induced by three different preservation media were quantified. A previous study by Vantipalli et al.^44^ investigated the effect of solutions with different tonicities on the cornea using OCT, applying a similar methodology to the first part of the present study and obtaining comparable results. The authors investigated the effects of hydration on central corneal thickness, resulting in a global description of the osmotic effects on the tissue. The present study employed high-resolution axial strains derived from OCE, which enabled a more detailed localized investigation of dynamic osmotic effects. We could examine the effects on the anterior and posterior cornea separately, providing new information on osmotic diffusion effects. While PBS alone caused swelling across the entire corneal thickness, varying the concentration of Dextran by 2% had distinct effects on the porcine cornea: MeM Dextran 7% solution caused a de-swelling effect on the anterior surface, while MeM Dextran 5% solution was almost isotonic. Our results showed that the deswelling of the tissue induced by MeM Dextran 7% solution in the anterior cornea was accompanied by swelling of the posterior cornea, which is consistent with the results of Lawman et al. on a human corneal sample without endothelium^25^. Although only the anterior stroma was exposed to the preservation medium, we observed different posterior strain under different conditions. This finding suggests that the forced deformation of the anterior stroma also leads to a change in the posterior strains, which could be explained by the migration of the fluid towards the posterior side of the cornea. Overall, the conditions investigated here show the strongest effects on the anterior half of the corneal stroma, while the posterior stroma generally tends to swell, with the exception of the MeM Dextran 5% condition, suggesting that not only endothelial cell death but also the osmotic state of the anterior cornea affects posterior corneal swelling.

Once the osmotically induced mechanical changes in the cornea were quantified using OCE, the set-up was used to evaluate the dynamic mechanical mechanism underlying CXL. To ensure that the system quantified a dynamic effect induced directly by CXL, rather than the osmotic swelling induced by the hydration media, three control groups were included, each lacking at least one of the element required to initiate CXL. No significant differences were found between the different control groups in terms of axial deformations induced during the treatment, with the cornea swelling almost constantly throughout the duration of the experiment. The CXL group, on the other hand, showed a shrinking of the anterior stroma that started after the start of the UV irradiation, progressively reaching a depth of 200 µm after ten minutes. The shrinking of the anterior cornea persisted for at least thirty minutes after the end of the CXL treatment. The posterior stroma showed no significant differences between CXL and the control groups, which is consistent with the fact that CXL only occurs in the first 300 µm of the stroma. The addition of 5% Dextran to the riboflavin solution would probably have reduced posterior corneal swelling, as indicated by the results of the first part of this study. However, our primary aim was to investigate the dynamic effects of CXL in a setting similar to the clinical one, and not to prevent osmotic diffusion during treatment. The inclusion of different control conditions did still allow to isolate the mechanical effect induced by CXL treatment. Considering the promising results of this study, this method could be further utilized to investigate the dynamic effects of riboflavin solutions with different tonicities.

The shrinkage of the anterior stroma induced by the CXL treatment could be attributed to two distinct mechanisms: on the one hand, the deformations that the tissue normally experiences when being exposed to the irrigation solution (PBS or RIBO) could be hindered by the fact that the tissue becomes increasingly stiff due to the newly formed cross-links. In other words, the osmotic pressure that caused the cornea to swell in the control groups would not produce enough osmotic stress on a stiffer cornea to make it swell. Since swelling of the anterior part is prevented by its stiffening, and the posterior part swells due to the absorbed riboflavin solution, the posterior part of the cornea could therefore lead to compression of the anterior stroma. Since the cornea was hydrated until the end of the experiments, the mechanism just described could explain why the shrinkage continued during up to 30 min after the treatment, while the radicals triggering the CXL reaction would disappear a few seconds the irradiation system was turned off. On the other hand, our results could indicate an active tissue shrinkage in the stroma due to a different molecular arrangement resulting from the newly formed cross-links. Although both hypotheses can explain the observed behavior, further studies are needed to investigate, for example, the effect of CXL at different osmotic pressures, or to evaluate the dynamic change in the molecular composition of the extracellular matrix.

**Figure 6:**
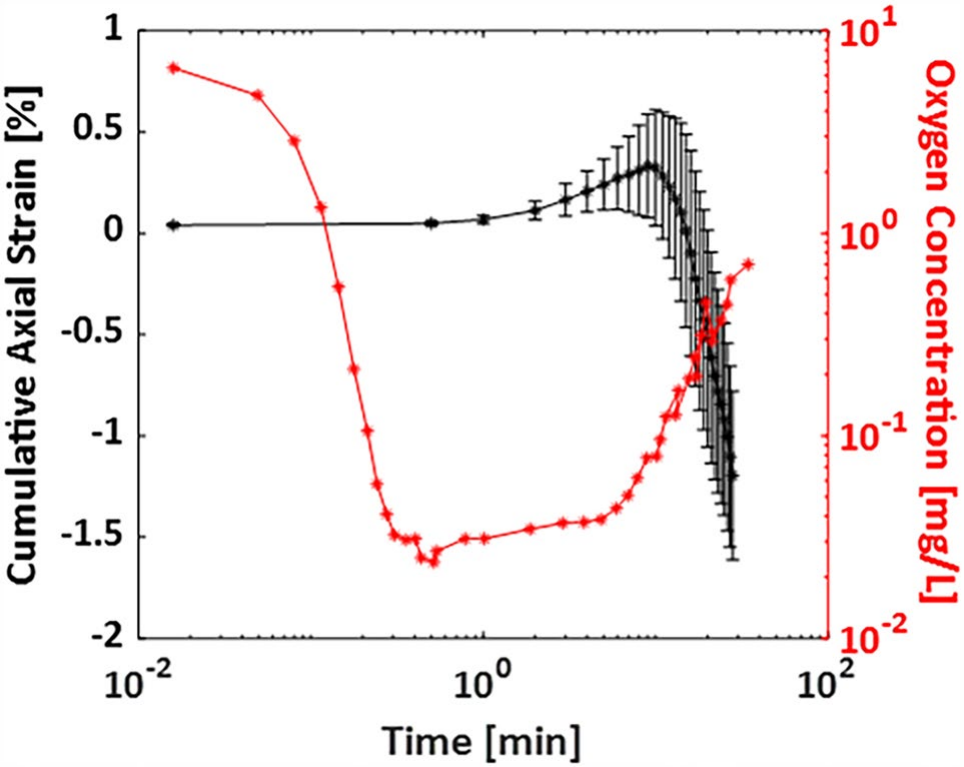
Oxygen consumption during CXL (red curve) as reported by Kamaev et colleagues ^36^ superposed to the induced mechanical axial strain induced during CXL measured by OCE (black curve). The x-axis is plotted on a logarithmic scale. OCE data are expressed as mean ± standard deviation.

The mechanism of oxygen consumption in CXL with an irradiance of 3 mW/cm^2^ was described in detail by Kamaev and colleagues^36^, who analyzed the time-dependent concentration of dissolved oxygen under a 100 µm corneal flap during treatment. In Fig. 6 we compare the oxygen concentration curve reported in Kamaev study with the active mechanical behavior of the tissue during CXL. The latter curve was obtained by subtracting the cumulative axial strain of the UV-only control group from the strain in the CXL group, both averaged over the anterior 200 µm. Interestingly, we found that during the first ten minutes, corresponding to a Type-I photochemical mechanism in which little oxygen is consumed by the reaction, the difference in mechanical strain between the CXL and control groups was almost zero. After this period, oxygen level begins to increase again, and axial strain becomes negative, indicating shrinkage of the anterior corneal region. This qualitative comparison, despite important limitations such as the different portion of anterior stroma considered in the two experiments (an average of the first 200 µm versus a corneal flap extracted in the first 100 µm) shows that the pattern of mechanical stiffening follows oxygen consumption during CXL, at least in the first 200 µm. This result is in agreement with previous literature reporting a strong dependence on oxygen availability^46^, concluding that the type-II reaction is probably the driving force of tissue stiffening. To further investigate and validate this hypothesis, the current strain measurement technique should be combined with oxygen measurements to relate the oxygen kinetics to the mechanical deformations of the sample.

The main limitation of the present study is the inadequate control of humidity and temperature during the measurement. Although the measurements were carried out at room temperature, the laboratory in which the measurements were taken is equipped with an air conditioning system that controls both humidity and temperature. Therefore, there were only slight fluctuations in these parameters during the measurements. For validation, osmotic diffusion could be independently quantified using fluorescent markers^47^. Since this technique was not available to us, the present experimental setup lacked a calibration of osmotic diffusion, hence, restricting the observations to relative comparisons. Another limitation was the use of porcine corneas instead of human samples, which are characterized by a different geometry and collagen fiber distribution in the stroma. Different sample species may influence the osmotic response to reach isotonicity, but the general trends observed in this study remain similar for human samples. Ideally, both the UV lamp and the OCT would have been placed perpendicular to the cornea. However, using a beam splitter would have induced dispersion artifacts in the OCT images. Therefore, instead the UV light was tilted, resulting in reduced energy and CXL effect in the periphery of the irradiated region. As the measured axial strain values were averaged across a relatively small 2 mm^2^ region that did not include peripheral areas, the average mechanical response is still representative of the UV energy applied. Finally, with the current setup, it was not possible to measure the force exerted on the tissue by osmotic gradients, which would have allowed a more robust analysis of the mechanical changes, including the stresses and therefore would have allowed an estimate of the mechanical properties of the tissue.

Three main results are presented in this paper: (i) OCT in combination with elastography successfully quantifies the dynamic osmosis-induced mechanical deformations of the cornea throughout its thickness; (ii) MeM Dextran 5% solution is a suitable preservation medium to keep tissue hydration stable with minimal osmotic effects; (iii) CXL treatment induces shrinkage of the anterior stroma, that persists for 30 min (and possibly longer) after the end of UV irradiation.

### Supplementary Information

Supplementary Figure S1.

## Data Availability

The data that support the findings of this study will become publicly available upon publication of the article in Zenodo at 10.5281/zenodo.12166457.
