# Supplementary material for: Dynamic evaluation of corneal cross-linking and osmotic diffusion effects using optical coherence elastography
Source: Sci Rep. 2024 Jul 18;14:16614. doi: 10.1038/s41598-024-67278-1 (PMC11258322; doi:10.1038/s41598-024-67278-1)

Supplementary Materials

Dynamic evaluation of corneal cross-linking and osmotic diffusion effects using optical coherence elastography

Authors: Matteo Frigelli[1], Philippe Büchler[1], *Sabine Kling[1]

[1]ARTORG Center for Biomedical Engineering Research, University of Bern, Switzerland

Corresponding Author:
sabine.kling@unibe.ch

Supplementary Figure S1: Dynamic quantification of depth-dependent effect of the different control treatments in the cornea. Left column PBS-only group, central column RIBO-only group, right column UV-only group. A-C) structural image of the central region, averaged across 480µm, as a function of time; D-F) corresponding axial strain maps; G-I) axial strain variation across the depth of the sample at minute 20 (black line), 50 (red lined) and 80 (blue line) for the considered groups. The first two rows show representative samples, while the last row shows the average profile across n=6 samples for the considered groups.

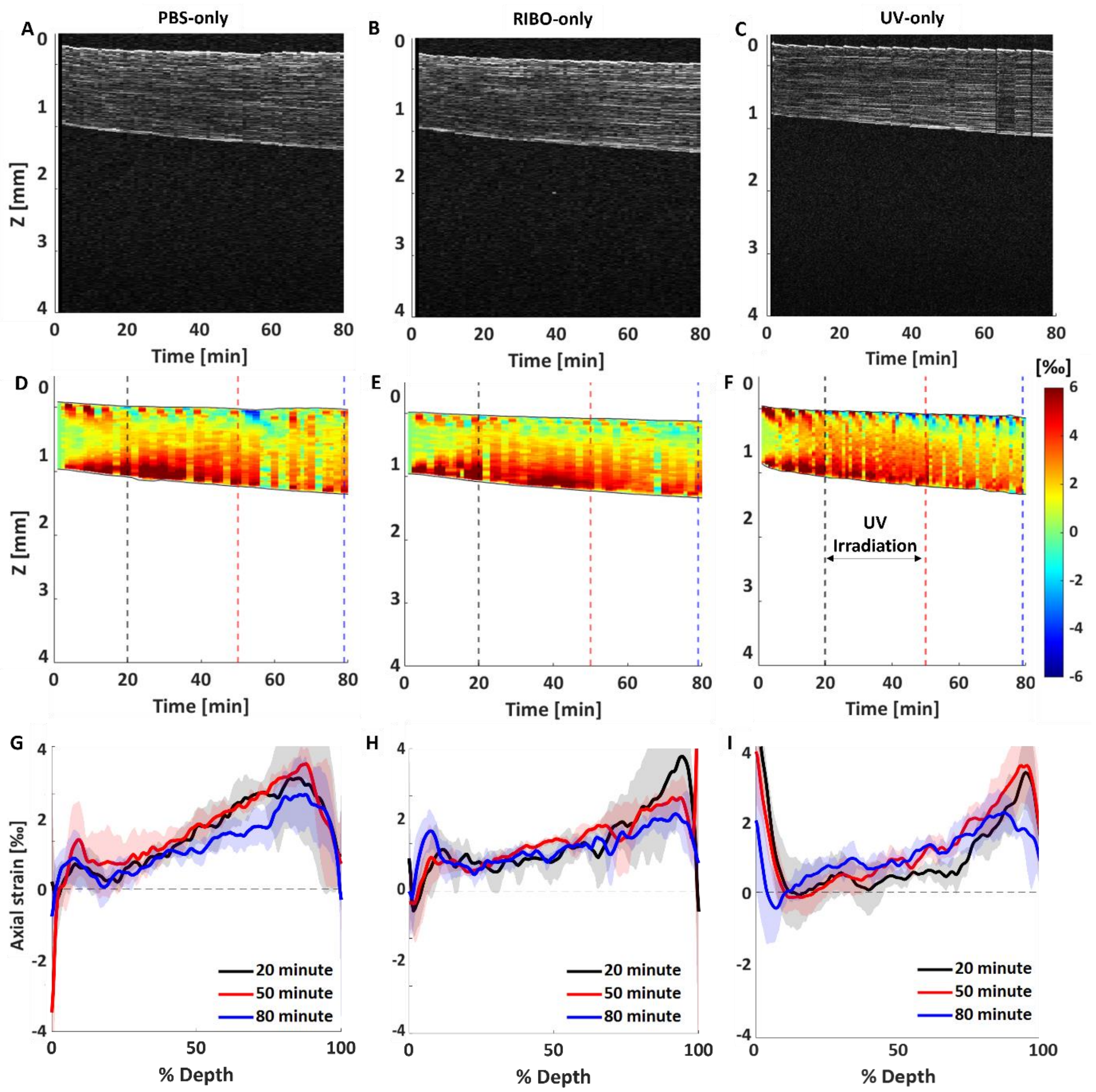

Supplement: Supplementary file 1 — Supplementary Figure S1. [file 41598_2024_67278_MOESM1_ESM.pdf]
